# Molecular Docking of Potential Inhibitors for Influenza H7N9

**DOI:** 10.1155/2015/480764

**Published:** 2015-03-15

**Authors:** Zekun Liu, Junpeng Zhao, Weichen Li, Xinkun Wang, Jingxuan Xu, Jin Xie, Ke Tao, Li Shen, Ran Zhang

**Affiliations:** Medical College, Hunan Normal University, Changsha, Hunan 410013, China

## Abstract

As a new strain of virus emerged in 2013, avian influenza A (H7N9) virus is a threat to the public health, due to its high lethality and pathogenicity. Furthermore, H7N9 has already generated various mutations such as neuraminidase R294K mutation which could make the anti-influenza oseltamivir less effective or ineffective. In this regard, it is urgent to develop new effective anti-H7N9 drug. In this study, we used the general H7N9 neuraminidase and oseltamivir-resistant influenza virus neuraminidase as the acceptors and employed the small molecules including quercetin, chlorogenic acid, baicalein, and oleanolic acid as the donors to perform the molecular docking for exploring the binding abilities between these small molecules and neuraminidase. The results showed that quercetin, chlorogenic acid, oleanolic acid, and baicalein present oseltamivir-comparable high binding potentials with neuraminidase. Further analyses showed that R294K mutation in neuraminidase could remarkably decrease the binding energies for oseltamivir, while other small molecules showed stable binding abilities with mutated neuraminidase. Taken together, the molecular docking studies identified four potential inhibitors for neuraminidase of H7N9, which might be effective for the drug-resistant mutants.

## 1. Introduction

As the WHO reported on June 27, 2014, there were 450 laboratory-confirmed cases of avian influenza A (H7N9) virus human infection including 165 deaths (http://www.who.int). Most patients presented with severe pneumonia, acute respiratory distress syndrome (ARDS), and multiple organ failure [1, 2]. Sequence analysis indicated that H7N9 gene descended from avian species, and what is more, the other six genes originated from H9N2 [1–3]. Since the H7N9 virus could bind to both the avian and human receptors, the potential risk of spreading between avian species and human was highly increased [2]. Previously, it was observed that the Asp701Asn mutation in PB2 and the Ser31Asn mutation in hemagglutinin increased the adaptability to mammals, and the Ser31Asn mutation of M2 contributed at least partly to the resistance of antivirus drugs including amantadine and rimantadine [3, 4]. These mutations made the treatment of H7N9 virus infected patients more difficult and increased the threats to public health. The neuraminidase inhibitor oseltamivir was reported to be effective and safe for therapy of influenza A [5] and employed in a number of H7N9 cases. Although a number of cases were cured [6, 7], oseltamivir was still invalid for a part of cases and the efficiency is still debatable [8, 9]. Recently, Wu et al. solved the structures for neuraminidases of A/Anhui/1/2013 (N9, R294) and A/Shanghai/1/2013 (N9, K294), which showed that the mutation of R294K could induce conformation changes and generate oseltamivir resistance through interfering with the binding of oseltamivir carboxylate [10]. Furthermore, the authors proposed that the drug resistance caused by mutation of R294K in H7N9 was more serious than that caused by mutation of H274Y in H7N1 [10]. Taken together, the development of new inhibitors for mutated neuraminidase is urgently needed.

Recently, molecular modeling and computational chemistry based computer-aided drug design provided great help for modern drug development [11]. Software programs such as AutoDock were widely used to search potential inhibitor for protein targets [12]. Previously, traditional Chinese medicine has been demonstrated to have antivirus ability according to the clinical data, and a number of small molecules such as quercetin, chlorogenic acid, oleanolic acid, and baicalein were regarded as active molecules [13]. For example, quercetin could serve as the inhibitor of Bcl-2 and Bcl-xL through binding with the BH3 domain, which promoted cancer cell apoptosis [14, 15]. Chlorogenic acid could protect the ApoE knockout mice against atherosclerosis through accelerating the cholesterol efflux from macrophages [16, 17]. Baicalein was identified as the inhibitor of neuraminidase for pandemic 2009 H1N1 and also inhibited interleukin-1 beta and TNF-alpha mediated inflammation [18, 19]. Through inducing autophagy, oleanolic acid could inhibit the proliferation and invasiveness in K-ras transformed cells and induce Nrf-2 related antioxidant to reduce ethanol-induced liver injury [20, 21]. However, whether these small molecules could inhibit the neuraminidase of H7N9 is still unclear.

In this study, we employed the strategy of molecule docking to explore potential H7N9 neuraminidase inhibitors from small molecules including quercetin, chlorogenic acid, oleanolic acid, and baicalein, which were bioactive components in traditional Chinese medicine. It was observed that these small molecules showed high binding energies with neuraminidases from A/Anhui/1/2013. The binding energies for these small molecules were comparable with oseltamivir, which indicated that the molecules were potential inhibitors for neuraminidase. Furthermore, the results showed a weak binding energy between oseltamivir and neuraminidase from A/Shanghai/1/2013, which was consistent with the oseltamivir resistance of H7N9 A/Shanghai/1/2013. However, the four molecules quercetin, chlorogenic acid, oleanolic acid, and baicalein showed robust binding energies with neuraminidase from A/Shanghai/1/2013. Taken together, these molecules were potential neuraminidase inhibitors which might be helpful for anti-influenza drug development.

## 2. Materials and Methods

### 2.1. Molecular Structure Preparing

To perform the molecular docking between neuraminidase and potential inhibitors, we employed the H7N9 neuraminidase structures of A/Anhui/1/2013 (PDB code: 4MWQ) and A/Shanghai/1/2013 (PDB code: 4MWW), which were solved by Wu et al. at the resolutions of 2.00 Å and 1.90 Å, respectively [10]. The PDB files for these proteins were downloaded from the RCSB Protein Data Bank (available at http://www.rcsb.org) [22]. The water and nonprotein molecules in the PDB files were removed, while only neuraminidase monomer structures were reserved. All the visualizations of molecular structures were performed with PyMOL, which is state-of-the-art software, to present the supercomplexes of proteins and small molecules (Version 1.7.2, available at http://pymol.org) [23]. Four small molecules including quercetin, chlorogenic acid, oleanolic acid, and baicalein from traditional Chinese medicine were selected for molecular docking based on the following principles: (1) the molecules should be identified from traditional Chinese medicine; (2) the molecules should have been studied previously and be commercially available. The structures in mol2 file format of the five small molecules, including oseltamivir carboxylate (ZINC03929509), quercetin (ZINC03869685), chlorogenic acid (ZINC02138728), oleanolic acid (ZINC03785416), and baicalein (ZINC03943903), were retrieved from the ZINC database, which is a free public resource for ligand molecules (available at http://zinc.docking.org) [24, 25].

### 2.2. Molecular Docking

In this study, we employed AutoDock software (Version 4.2, available at http://autodock.scripps.edu) to perform molecular docking. The AutoDock is an automated docking program designed for prediction of the binding among small molecules such as substrates or drug candidates and the receptor with known 3D structure [12]. AutoDockTools software (Version 1.5.6, also available at http://autodock.scripps.edu), the graphical user interface (GUI) program for setting up AutoDock software, was used to prepare the procedures of docking [12]. The volume of the grid box was set as 60 × 50 × 60 with the default 0.375 Å spacing, and the number of docking runs was set as 100. The neuraminidase structures were used as the receptor for docking with the small molecules. Since the active sites of neuraminidase in influenza A were highly conserved, the docking pocket was set as the eight key residues including Arg-118, Asp-151, Arg-152, Arg-224, Glu-276, Arg-292, Arg-371, and Tyr-406 [26–28]. The chemoinformatic analyses of potential toxicities and promiscuities for the five small molecules were performed with FAF-Drugs3 software (available at http://fafdrugs3.mti.univ-paris-diderot.fr) [29].

## 3. Result and Discussion

### 3.1. Characterizing the Structures of the Small Molecules for Molecular Docking

**Figure 1:**
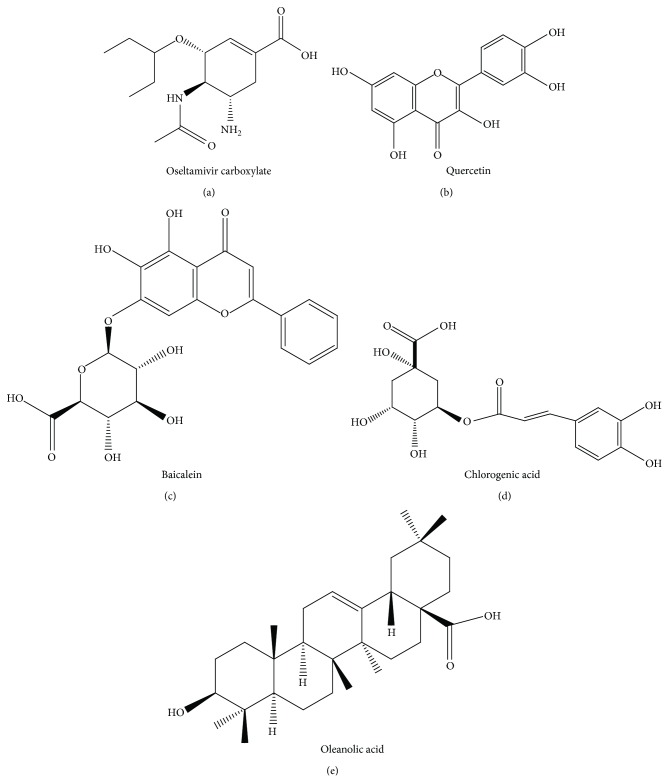
The molecular structures for the five small molecules: (a) oseltamivir carboxylate; (b) quercetin; (c) baicalein; (d) chlorogenic acid; (e) oleanolic acid.

In this study, five small molecules including oseltamivir carboxylate (Figure 1(a)), quercetin (Figure 1(b)), baicalein (Figure 1(c)), chlorogenic acid (Figure 1(d)), and oleanolic acid (Figure 1(e)) were employed for docking with neuraminidase to explore the potential inhibitor. Previously, it was observed that the oseltamivir phosphate is the prodrug without antivirus activity while its product of metabolism oseltamivir carboxylate is active [30]. Thus, in this study, the oseltamivir carboxylate was employed for molecular docking. The chemoinformatic analyses were performed with the FAF-Drugs3 software [29] to further understand the properties of the small molecules. Since the hydrogen bonds (H-bonds) were critical for the interactions and binding affinity between the protein and small molecules, we analyzed the H-bond donors and acceptors of these small molecules. It was observed that oseltamivir carboxylate and quercetin, chlorogenic acid and baicalein have five and six H-bond donors, respectively, while oleanolic acid has two (Table 1). Baicalein has 11 H-bond acceptors, while chlorogenic acid, quercetin, oseltamivir carboxylate, and oleanolic acid have 9, 7, 6, and 3 H-bond acceptors, respectively (Table 1). These results showed that quercetin, chlorogenic acid, oleanolic acid, and baicalein had the potential to form H-bonds with proteins. Furthermore, the potential toxicities and promiscuities were analyzed by the FAF-Drugs3 software [29]. It was observed that baicalein, chlorogenic acid, quercetin, and oseltamivir carboxylate have low toxicities and promiscuities according to the threshold of log⁡⁡*P* < 3 and tPSA > 75 (Table 1). Furthermore, oleanolic acid has the log⁡⁡*P* of 7.49 and tPSA of 60.36 (Table 1). However, oleanolic acid was proved as a drug for human liver disorders treatment [31]. Thus, it seemed that four small molecules had limited toxicities and promiscuities and were proper for further drug development.

### 3.2. Molecular Docking between NA and Small Molecules

**Figure 2:**
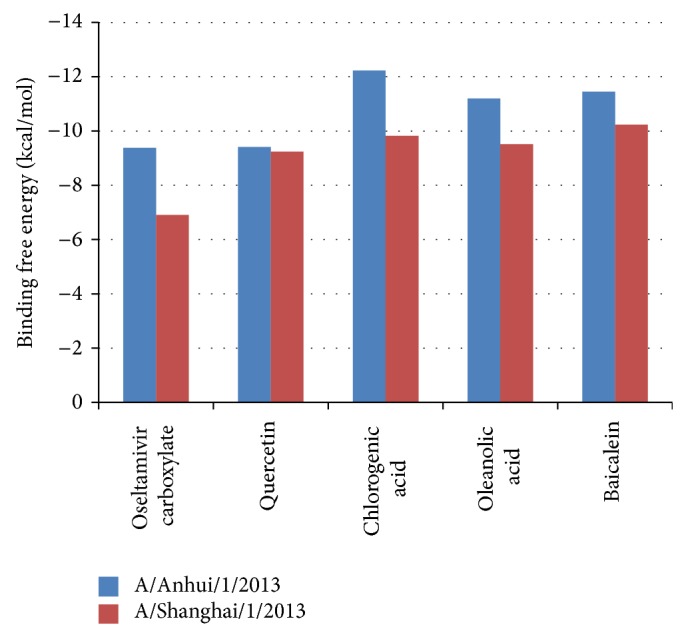
The binding free energies for small molecules and neuraminidase from H7N9 A/Anhui/1/2013 and A/Shanghai/1/2013.

The molecular docking was performed among the neuraminidases from H7N9 A/Anhui/1/2013 and A/Shanghai/1/2013. Oseltamivir carboxylate could serve as a control to evaluate the neuraminidase binding ability of other molecules. The conformation variations, binding energies, and inhibition constants for the molecular docking among neuraminidase and small molecules were presented in Figure 2 and Tables 2 and 3. The calculated root mean square deviations (RMSDs) indicated that the conformation variations among neuraminidases binding with different small molecules were limited, while A/Shanghai/1/2013 neuraminidase had moderate large variations (Tables 2-3). It was observed that the A/Anhui/1/2013 neuraminidase binding energies for oseltamivir carboxylate and quercetin were almost the same, which were −9.38 kcal/mol and −9.41 kcal/mol, respectively, while chlorogenic acid, oleanolic acid, and baicalein have higher binding energies as −12.23 kcal/mol, −11.20 kcal/mol, and −11.45 kcal/mol, separately. The inhibition constants calculated for oseltamivir carboxylate, quercetin, chlorogenic acid, oleanolic acid, and baicalein were 132.26, 126.87, 1.09, 6.19, and 4.02 nM, respectively (Table 2). For the A/Shanghai/1/2013 neuraminidase, the binding energies for oseltamivir carboxylate, quercetin, chlorogenic acid, oleanolic acid, and baicalein were −6.91 kcal/mol, −9.24 kcal/mol, −9.82 kcal/mol, −9.51 kcal/mol, and −10.32 kcal/mol. It was obvious that oseltamivir carboxylate showed weaker binding ability for A/Shanghai/1/2013 neuraminidase than A/Anhui/1/2013, which was consistent with the observation that A/Shanghai/1/2013 has resistance for oseltamivir [10]. Other molecules including chlorogenic acid, oleanolic acid, and baicalein also presented the decrease of binding energies; quercetin showed robust binding abilities for neuraminidases from H7N9 A/Anhui/1/2013 and A/Shanghai/1/2013. The inhibition constants calculated for oseltamivir carboxylate, quercetin, chlorogenic acid, oleanolic acid, and baicalein were 8.59*E* + 03, 168.62, 63.6, 106.11, and 27.11 nM, respectively (Table 2), which also indicated that H7N9 neuraminidase had significant oseltamivir resistance. Furthermore, the detailed results for the top 3 scored poses for each small molecule were presented in Supplementary Table S1 (see Supplementary Table S1 in Supplementary Material available online at http://dx.doi.org/10.1155/2015/480764).

### 3.3. Structural Insights of the Docking Complex

**Figure 3:**
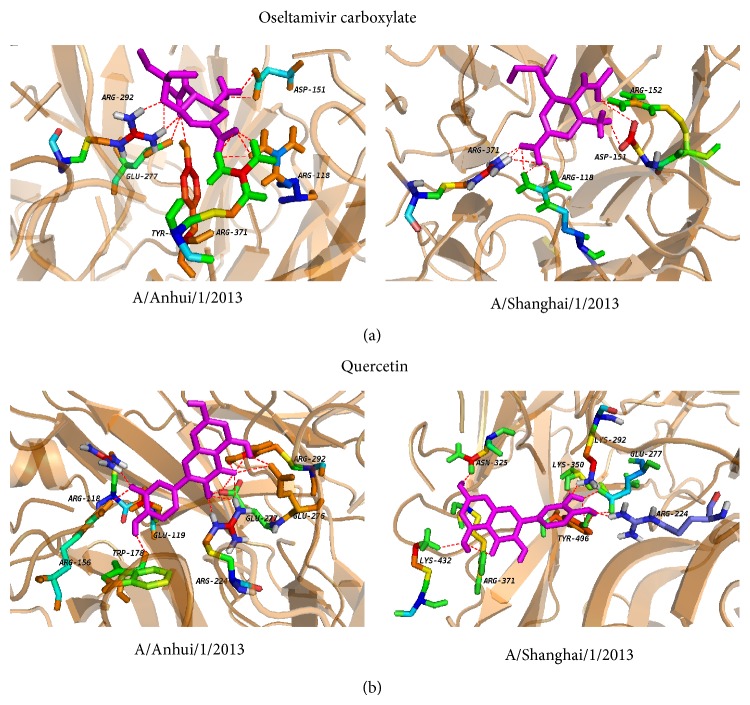
The local structure of the docking complexes for small molecules and neuraminidase: (a) oseltamivir carboxylate and (b) quercetin.

**Figure 4:**
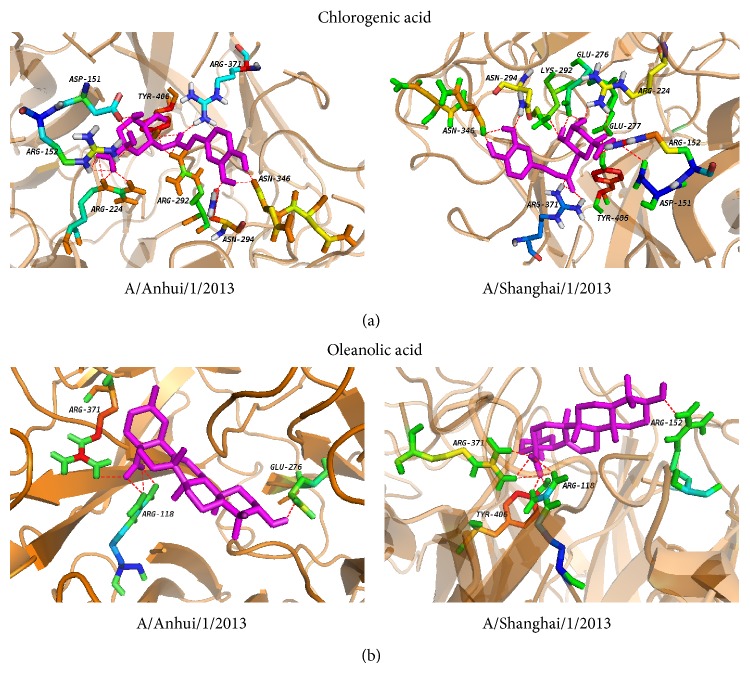
The local structure of the docking complexes for small molecules and neuraminidase: (a) chlorogenic acid and (b) oleanolic acid.

**Figure 5:**
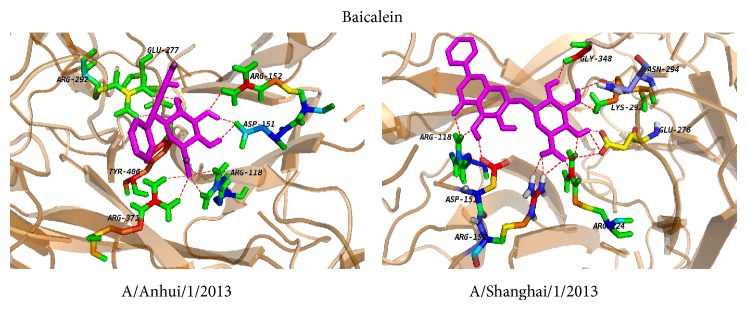
The local structure of the docking complexes for baicalein and neuraminidase.

To further dissect the interaction between neuraminidase and the small molecules, the structures in complex derived from the best dock results with neuraminidase for oseltamivir carboxylate (Figure 3(a)), quercetin (Figure 3(b)), chlorogenic acid (Figure 4(a)), oleanolic acid (Figure 4(b)), and baicalein (Figure 5) derived from molecular docking were shown. From the molecule docking among A/Anhui/1/2013 neuraminidase and small molecules, it was observed that there were 18 H-bonds in the neuraminidase-chlorogenic acid complex (Figure 4(a)), while quercetin (Figure 3(b)) has 14 H-bonds with neuraminidase. Oseltamivir carboxylate (Figure 3(a)) and baicalein (Figure 5) both have 12 H-bonds with neuraminidase, while oleanolic acid (Figure 4(b)) has 5. The H-bond residues for neuraminidase from A/Anhui/1/2013 and A/Shanghai/1/2013 were provided in Tables 4 and 5, respectively. These results indicated that chlorogenic acid, quercetin, and baicalein could intensively bind neuraminidase through H-bonds. For A/Shanghai/1/2013 neuraminidase, chlorogenic acid could form 17 H-bonds (Figure 4(a)), which indicated that chlorogenic acid has high potential of neuraminidase inhibition. It was interesting that the H-bonds for baicalein (Figure 5) and oleanolic acid (Figure 4(b)) were increased to 15 and 8 for A/Shanghai/1/2013 neuraminidase compared to those from A/Anhui/1/2013, while there were 9 and 11 H-bonds for oseltamivir carboxylate (Figure 3(a)) and quercetin (Figure 3(b)), respectively. These results indicated that the oseltamivir resistance caused by R294K mutation for neuraminidase in A/Shanghai/1/2013 might be generated from the H-bond loss, while other molecules might overcome the resistance. Furthermore, other known inhibitors of neuraminidase including zanamivir, peramivir, and laninamivir had similar structures and presented similar inhibition abilities for neuraminidase from A/Anhui/1/2013 [10]. However, they also showed significantly decreased inhibition for A/Shanghai/1/2013 [10]. Since the small molecules including quercetin, chlorogenic acid, oleanolic acid, and baicalein had longer structures and there was considerable room for improvements, we anticipated that they had great potential to overcome the drug resistance of H7N9 mutant.

## Supplementary Material

The Supplementary Material of Table S1 provide the detailed results of the top 3 scored poses for the complexes of neuraminidases from A/Anhui/1/2013 and A/Shanghai/1/2013 docking with each small molecule.

## Figures and Tables

**Table 1:** The chemoinformatic analyses of the small molecules with FAF-Drugs3 software.

	MW	log⁡⁡*P*	tPSA	Rotatable bonds	HB donors	HB acceptors	Oral bioavailability (VEBER)	Oral bioavailability (EGAN)
Oseltamivir carboxylate	285.36	−1.87	106.1	6	5	6	Good	Good
Quercetin	302.24	2.17	134.2	1	5	7	Good	Good
Chlorogenic acid	354.31	−0.42	167.6	5	6	9	Good	Good
Oleanolic acid	456.7	7.49	60.36	1	2	3	Good	Good
Baicalein	446.36	1.11	190	4	6	11	Good	Good

**Table 2:** The energies for the binding between the small molecules and A/Anhui/1/2013 neuraminidase.

Molecules	RMSD ($\overset{´}{Å}$)	(vdW + Hbond + desolv) energy (kcal/mol)	Electrostatic energy (kcal/mol)	Total internal energy (kcal/mol)	The best docking energy (kcal/mol)	Inhibition constant (nM)
Oseltamivir carboxylate	—	−7.24	−3.46	−11.47	−9.38	132.26
Quercetin	0.38	−6.69	−1.27	−11.2	−9.41	126.87
Chlorogenic acid	0.33	−10.24	−3.11	−15.21	−12.23	1.09
Oleanolic acid	0.81	−6.18	−2.2	−11.79	−11.2	6.19
Baicalein	0.38	−8.69	−3.3	−18.95	−11.45	4.02

**Table 3:** The energies for the binding between the small molecules and A/Shanghai/1/2013 neuraminidase.

Molecules	RMSD ($\overset{´}{Å}$)	(vdW + Hbond + desolv) energy (kcal/mol)	Electrostatic energy (kcal/mol)	Total internal energy (kcal/mol)	The best docking energy (kcal/mol)	Inhibition constant (nM)
Oseltamivir carboxylate	—	−4.22	−3.94	−9	−6.91	8.59*E* + 03
Quercetin	1.09	−6.17	−1.34	−11.03	−9.24	168.62
Chlorogenic acid	0.83	−6.98	−2.42	−12.8	−9.82	63.6
Oleanolic acid	0.42	−4.36	−4	−10.11	−9.51	106.11
Baicalein	0.73	−6.57	−3.28	−16.23	−10.23	27.11

**Table 4:** The residues for the H-bonds between the small molecules and A/Anhui/1/2013 neuraminidase.

Molecules	H-bond residues
Oseltamivir carboxylate	ARG-292, ASP-151, GLU-277, ARG-118, ARG-371, and TYR-406
Quercetin	ARG-118, GLU-119, GLU-277, ARG-292, GLU-276, ARG-156, TRP-178, and ARG-224
Chlorogenic acid	ASP-151, ARG-152, ARG-224, ARG-292, ASN-294, ASN-346, ARG-371, and TYR-406
Oleanolic acid	ARG-118, GLU-276, and ARG-371
Baicalein	ARG-292, GLU-277, ARG-152, ASP-151, ARG-118, ARG-371, and TYR-406

**Table 5:** The residues for the H-bonds between the small molecules and A/Shanghai/1/2013 neuraminidase.

Molecules	H-bond residues
Oseltamivir carboxylate	ARG-118, ASP-151, ARG-152, and ARG-371
Quercetin	ARG-224, GLU-277, LYS-292, LYS-350, ASN-325, ARG-371, LYS-432, and TYR-406
Chlorogenic acid	ASN-346, ASN-294, LYS-292, GLU-276, ARG-224, GLU-277, ARG-152, ASP-151, TYR-406, and ARG-371
Oleanolic acid	ARG-118, ARG-152, ARG-371, and TYR-406
Baicalein	ARG-151, ARG-118, ARG-152, ARG-224, GLU-276, LYS-292, ASN-294, and GLY-348
